# An Article on Malignant Diphtheria, with Report of Eleven Cases

**Published:** 1865-01

**Authors:** D. W. Young

**Affiliations:** Geneva, Illinois


### CHICAGO MEDICAL EXAMINER.

N. S. DAVIS, M.D., Editor.

VOL. VI. JANUARY, 1865. NO. 1.

### Original Contributions.

### ARTICLE I.

### AN ARTICLE ON MALIGNANT DIPHTHERIA, WITH REPORT OF ELEVEN CASES.

By D. W. YOUNG, M.D., of Geneva, Illinois.

Read before the Fox River Valley Medical Association, at Geneva, Illinois, Jan. 2, 1865.

Mr. President, and Gentlemen of the Fox River Valley Medical Association:

I most respectfully ask your indulgence for a few moments, while I read the following brief history of cases of Diphtheria that have come under my observation since our last meeting.

I do not offer them expecting to present anything new or novel, either in theory or practice. I simply offer them for the purpose of adding my mite to the general record of the medical profession upon this subject, hoping thereby to induce such discussion as shall eventually produce a correct pathology and successful treatment of this terrible disorder.

Case I.—Mrs. B., a stout, robust German lady, of bilious temperament, and previous good health, aged 23 years, married, and mother of one child aged 14 months, was attacked with chills, followed by fever and sore throat, about the 12th of August, 1864. At first, she resorted to the usual domestic remedies, but failing to obtain relief, called Dr. ——, her usual medical attendant. He attended her for several days, when, for some reason, she became dissatisfied with him and sent for me. I first visited her on the 19th of the month, and found her with considerable fever, small, rapid pulse, and extreme prostration. There was quite a good deal of swelling externally about the angles of the jaw, face, and neck. Upon inspecting the throat and mouth, I found the tonsils swollen and inflamed, and the left one and a large portion of the roof of the mouth, upon the left side, thickly covered with diphtheritic exudation. There was but little fetor, much less than I had usually found in these cases. The membrane was peculiarly tough, and closely adherent. The swelling of the tonsils was so extensive as to materially interfere with respiration and deglutition. She was very much prostrated, and seemed greatly alarmed concerning herself.

I removed as much of the exudation as I could conveniently, and then applied freely, with a surgeon's camel-hair brush, a saturated solution of nitrate of silver. I ordered her to steam her throat often and thoroughly with the fumes of strong vinegar and sage; also, to rub the skin externally freely with kerosene, and then apply a thin slice of salt pork. I left her quiniæ sulph. grs. xxx, Doveri pulv. grs. xv, divided into six powders, and gave one every four hours; also, 20 drops of the muriated tincture of iron every four hours. Made a strong solution of nutgalls and alum to use as a gargle.

*August* 20.—Mrs. B. no better; had spent a poor night. She looked and felt very bad. Some fever. Pulse 120, small, and quick; swelling about neck and face increasing, respiration labored, deglutition difficult and painful, exudation abundant and tenacious. Immediately in front of the left tonsil, extending up to and around the uvula was a deep, ragged, bad-looking ulcer. I again removed as much of the membrane as I could conveniently, then cauterized the ulcer thoroughly with the solid stick of nitrate of silver, and advised a continuance of the steaming and garling. Continued the other treatment.

*Aug.* 21, 9 *o'clock A.M.*—Patient looked bad. She said she was worse, and could not live long. She and her friends were greatly alarmed concerning her condition. External swelling about the same. The kerosene and salt pork had produced a free eruption. Respiration still labored, deglutition very difficult and painful. When she attempted to swallow, a portion of the fluids were rejected through the nose. Pulse 123, and feeble, swelling of tonsils extreme, and both covered with exudation. The ulcer, spoken of at last visit, was about the same. She complained of being very tired and prostrate. Touched the ulcer again with the nitrate of silver, and continued all the other medicines. In consequence of her extreme prostration, I ordered her to have two table-spoonsful of bourbon whiskey every four hours.

*Aug. 22d,* 9 *o'clock A.M.*—Mrs. B. looked and felt much better. She had slept pretty well during the night, and felt more hopeful. Swelling about face and neck less, respiration and deglutition improving, pulse 118, slower and fuller, exudation not so profuse nor tenacious, no fetor, exudation peeled off quite easily in large flakes or chunks; the surface below was pale blue, and bled when touched; ulcer about the same, no deeper. Dissolved two drachms of kreosote in half an ounce of 95 per cent alcohol, and then washed the throat thoroughly with that mixture. Continued the other treatment, except substituting warm poultices for the salt pork, and advised the crowding of chicken broth, and other nourishment.

*Aug. 23d,* 10 *o'clock A.M.*—Patient improving in all respects except swallowing and talking. She had considerable pain when she swallowed, and a portion of the fluids were still rejected through the nose. She had also lost the power of articulation, and it was with the greatest difficulty that she could make us understand her wants. Throat looked much better, exudation disappearing, and ulcer healing. Again washed the throat with the kreosote mixture, and continued the other treatment as at last visit.

*Aug. 24th,* 10 *o'clock A.M.*—Mrs. B. improving rapidly in every respect except talking. Swelling was disappearing, and she swallowed better. Appetite improving, bowels regular, pulse 94, soft and fuller. Continued treatment.

*Aug. 25th,* 10 *o'clock A.M.*—Mrs. B. bolstered up in bed, feeling finely. Swelling and exudation had pretty much disappeared, articulation improving, but still very imperfect. Advised a continuance of the muriated tincture of iron and bourbon whiskey in reduced quantities and at longer intervals, for several days, and left her. Sept. 28th, her husband called at my office, *paid his bill*, and said she was doing nicely, all but talking, and that was improving. Oct. 15th, I met her at one of her neighbors, and she was quite fleshed up, and looked well. Her articulation was still very imperfect. From her friends I learn that her speech at this date is not fully restored, but improving.

CASE II.—*August 30th*, 1864, was called to see Willie W., a stout, fleshy German boy, aged six years, of good constitution and previous good health, who was attacked with chills on the day previous, and had some fever, and considerable swelling about the angles of the jaw, face, and neck. He complained a great deal of stiffness and soreness in the muscles of the neck and throat. Upon examining his mouth and throat, I found the tonsils largely swollen, and they and a portion of the roof of the mouth well covered with diphtheritic exudation. He had no appetite, and he said it pained him when he swallowed. Pulse 100 per minute, and quick, bowels had moved that morning, skin dry and hot, considerable thirst. I left him quiniæ sulph. grs. xv, pulvis Doveri, grs. v, divided into five powders, one to be given every four hours; also, 10 drops of muriated tincture of iron every four hours; steam the throat freely and frequently with the fumes of sour vinegar and sage, rub the skin about the angles of the jaw and the neck well with kerosene, and apply a thin slice of salt pork about the neck.

*Aug. 31st*, 11 *o'clock A.M.*—Willie no better. His mother said that he had passed an uneasy, restless night. He had not had any recurrence of chills since the first day. Fever subsiding a little, skin slightly moist, pulse 110 and very quick, swelling externally extending; the kerosene and pork have irritated the skin considerably, he had considerable difficulty in swallowing, tonsils and uvula well enveloped in exudation, very little fetor. Continued all the treatment of the previous day, and ordered an addition of a tablespoonful of bourbon whiskey every four hours.

*Sept.* 1*st*, 10 *o'clock A.M.*—Willie is decidedly better in all respects. Rested well last night, and sweat freely; swelling and exudation disappearing rapidly. Advised a continuance of the remedies for a few days longer, and discharged him.

CASE III.—*Sept.* 6*th*, 1864, was called to Nancy W., a stout, healthy looking girl, a sister to Willie, aged nine years, also of good constitution and previous good health, who had been complaining for several days. Yesterday she had a chill, which was followed with fever and sore throat. She had considerable swelling about the angles of the jaw, face, and neck, some fever, skin hot and dry, pulse 100, and quick, and also complained of stiffness and soreness in the muscles of the neck and throat. Upon examination, I found the tonsils swollen, fauces inflamed and covered with diphtheritic exudation. There was more fetor in her case than in the others. I left her quiniæ sulph. grs. xv, Doveri pulv. grs. vj, divided into five powders, with directions that one be taken every four hours; also ten drops of muriated tincture of iron in a tablespoonful of bourbon whiskey every four hours, steam the throat well and frequently with the fumes of strong vinegar and sage, and apply externally the kerosene and salt pork.

Willie was up and around, but very weak.

*Sept.* 7*th*, 11 *o'clock A.M.*—Nancy decidedly better; rested well last night, sweat freely, and was nearly free from fever; swelling decreasing, exudation less, pulse 94, and slower. Continued same treatment.

*Sept.* 8*th*, 10 *o'clock A.M.*—Improving in all respects; swelling and exudation nearly disappeared. Advised a continuance of the whiskey and iron for a few days longer, and discharged her.

CASE IV.—*October* 31*st*, 1864, was called to see M. H., a stout German boy, aged nine years, of good constitution and previous good health, and a cousin to the above-reported patients. His parents informed me that he had been very healthy and well up to Sunday, the day previous to my visit, when he first began to be indisposed. They said that he had attended the funeral of an older brother on Saturday, got slightly wet, and on Sunday complained of being chilly, headache, and sore throat. I found him with high fever, dry skin, swelled face and neck, and difficulty in swallowing, pulse 100 and quick, no appetite. Upon inspecting the mouth and throat, I found the tonsils swelled and inflamed, and studded with points of diphtheritic exudation; no fetor. Complained of a good deal of stiffness and soreness in the muscles of the neck and throat. I left him quiniæ sulph. grs. xv, Doveri pulv. grs. vj, divided into five powders, and ordered one to be given every four hours; also fifteen drops of the muriated tincture of iron every four hours, steam the throat thoroughly with fumes of strong vinegar and sage, and externally apply the kerosene and salt pork.

*Nov.* 1*st*, 9 *o'clock A.M.*—Patient no better. The swelling externally had increased decidedly, respiration labored, deglutition quite difficult, tonsils and uvula completely covered with exudation, pulse 110, quick but weaker. Washed the throat with the kreosote mixture, and continued the other treatment, adding a tablespoonful of bourbon whiskey every two hours.

*Nov.* 2*d*, 10 *o'clock A.M.*—No better; all the symptoms seemed aggravated. Swelling extensive, the mouth and throat literally filled with dirty-looking exudation, respiration and deglutition difficult, no fetor, skin cool, pulse 120 per minute, quick and feeble. He had taken everything but the whiskey, and had such an aversion to it that they could not get him to take it at all. Removed as much of the exudation as I could. The surface below the membrane looked blue, and bled freely, in consequence of which I made a solution of the persulphate of iron, and washed the throat freely with it. Continued the other treatment, and added fifteen drops of the syrup of the iodide of iron once in four hours, in place of the whiskey.

*Nov.* 3*d*, 10 *o'clock A.M.*—M. alive, but failing. Neck, face, and throat enormously swollen, respiration very difficult, deglutition almost impossible, a large portion of all fluids are rejected through the naries, roof of mouth, tonsils, uvula, and fauces thickly covered with ragged, filthy-looking membrane, no fetor. The patient had a peculiar, *sallow, lifeless, tallowy look.* Skin cool and dry. When he coughs or strangles, he throws up large pieces or chunks of membrane and flesh. Pulse 125 per minute, quick, and very feeble. They had attempted to give him the whiskey, but it strangled him, and they had to desist. I advised them to wrap him up in warm flannels, and make him as comfortable as circumstances would admit.

*Nov. 4th*, 10 *o'clock A.M.*—M. died last night, about 11 o'clock.

CASE V.—*November 2d.*—Had my attention called to Levi, a brother of M., aged 5 years, of good constitution, and previous good health, who had been complaining for several days; had a chill that morning, and had considerable fever and complained of sore throat. Pulse rapid; skin dry and hot. Upon examining the mouth and throat, I found his tonsils swollen and inflamed, but no exudation. I left him quiniæ sulph. grs. xij, Doveri pulvis, grs. iij, divided into six powders; one to be given every four hours. Also, ten drops of muriated tincture of iron every four hours. Ordered them to steam his throat freely with the fumes of strong vinegar and sage. Applied the kerosene and salt pork to the neck.

*November 3d*, 10 *o'clock A.M.*—Levi about the same. There was rather more swelling about the face and neck, and he now complained of stiffness and soreness of the muscles of the neck and throat. The tonsils were studded with points of exudation; no fetor. Brushed his tonsils and throat with a solution of the persulphate of iron; continued the other treatment, and added a tablespoonful of bourbon whiskey every two hours.

*November 4th*, 10 *o'clock A.M.*—Levi not so well; passed a bad night; I think probably he had been neglected somewhat in consequence of his brother's dying, and the friends having been engaged with him. The swelling about his face and neck had increased; respiration labored; deglutition difficult and painful; tonsils and roof of mouth well covered with membrane. Pulse, 115 per minute, and quick; skin cool, but dry; no fetor. Continued treatment.

*November 5th*, 10 *o'clock A.M.*—Levi about the same; swelling full as much; pulse 125, quick and feeble; respiration labored; deglutition very painful; exudation profuse, and began to peel off in large pieces; no fetor. The surface where the exudation peeled off looked pale and blue, and bleeds upon the slightest touch; he complained of being very tired; skin cool, and he began to have that pale, sallow, lifeless, tallowy look and expression. He had a great aversion to food, and it required great efforts to get him to take the broths or nourishment. Continued all the former treatment, and added fifteen drops of the syrup of iodine of iron every four hours.

*November 6th, 10 o'clock A.M.*—Levi better. There was less swelling and less exudation; pulse 110 per minute, and fuller; respiration improving; sleeps a great deal; bowels regular. Continued treatment.

*November 7th, 10 o'clock A.M.*—Levi improving slowly; he looked pale and prostrate; swelling subsiding; exudation decreasing; throat looks pale and flabby; pulse 108; sleeps most of his time; skin cool; bowels regular; washed his throat with the kreosote mixture, and continued other treatment.

*Nov. 8th, 10 o'clock A.M.*—Levi improving in all respects. Continued treatment.

*November 9th, 10 o'clock A.M.*—Levi doing well; swelling and exudation decreasing; appetite improving a little; still dull and sleepy; skin warmer, and better color; kerosene and pork have blistered the skin from ear to ear; applied a poultice and continued other treatment.

*November 10th.*—Being absent from the city, my friend, Dr. Woodworth, visited Levi for me; he thought him not quite so well; he was thoroughly covered with a peculiar erythematous redness simulating scarlatina rash, so much so that the Doctor apprehended it scarlatina supervening. There was an increased swelling of the tonsils, with redness of the fauces and uvula; there was also a small point of exudation upon the left tonsil, which he touched with the solid nitrate of silver; in consequence of the scarlatina-like appearance, the Doctor ordered him a warm bath, after which he be well rubbed all over with fresh lard; he also recommended that he have the dilute hydrochloric acid. Continued the stimulus and iron.

*November 11th, 10 o'clock A.M.*—Levi had a little more fever than when I saw him last, on the 9th; the redness spoken of by Dr. Woodworth had pretty much disappeared; there was a little more swelling and redness of the tonsils and fauces; no exudation; still sleepy; continued quinæ, iron, and stimulants.

*November 12th, 10 o'clock A.M.*—I think Levi improving, but very slowly; he presented a little more color and lifelike appearance; he was very prostrate and sleepy; very little swelling; no exudation; had taken his nourishment more willingly; bowels regular; pulse 103; slower and fuller; throat pale and flabby. Continued treatment.

*November 15th, 10 o'clock A.M.*—Levi doing well; continued treatment. This patient continued to improve, and finally got about after a very protracted convalescence.

CASE VI.—*November 7th*, 1864, was requested to examine Louisa, a fleshy little girl aged one year and four months; sister to M. and Levi, who had been quite fretful and feverish since yesterday; her mother said that she had never been sick since she was born; I found her with some fever and hot, dry skin; rapid pulse and hurried respiration. Upon inspecting the mouth and throat I found the tonsils swollen and red; fauces dry and red; no exudation; eyes suffused and red; bowels loose; wanted to nurse most of the time; prescribed her quinæ sulph. grs. xij, Doveri pulvis, grs. ij, in six powders, one to be given every four hours; also ten drops muriated tincture of iron every four hours; warm fomentation to the throat and neck.

*November 8th, 10 o'clock A.M.*—Louisa very bad; tonsils and fauces badly swollen and thickly covered with a peculiarly filthy looking, closely adhering exudation; respiration hurried; deglutition difficult; face and neck swelled; pulse 130; very quick; cross when moved; refused the breast; continued the quinine and muriated tincture; tried to have her swallow some bourbon whiskey, but it strangled her so that we had to desist; advised wine whey instead; this she retained readily; applied the kerosene and salt pork to the throat and neck, in place of the warm fomentations.

*November 9th, 10 o'clock A.M.*—Louisa no better; she looked bad, and began to have that same sallow, lifeless, tallowy appearance; external swelling extensive; she was now having a profuse discharge of a bloody, acrid matter, from the nostrils, which excoriated the upper lip and cheeks; respiration bad; deglutition very difficult; a large portion of fluids attempted to be swallowed were rejected by the nose; skin shiny and cool; pulse 130 per minute, and feeble; tonsils and fauces covered with nasty, ragged-looking exudation; washed the throat with the kreosote mixture, and continued other treatment; urged them to give her all the wine whey and chicken broth she would retain.

*November* 10*th.*—Being absent from the city, Dr. Woodworth saw her for me, and reported her failing; he did not deem it advisable to make any change in her treatment.

*November* 11*th,* 10 *o'clock A.M.*—Louisa no better; looked, and was, very bad; did not discover any particular change since my last visit; I think there was greater prostration. Continued same treatment, and added ten drops of the syrup of the iodide of iron every four hours.

*November* 12*th,* 10 *o'clock A.M.*—Louisa looked and appeared a little better; she had had a new and more experienced nurse for a day or two, and had been thoroughly cleansed, washed and changed since my last visit, and, as a consequence, looked and appeared improved. There was not quite as much swelling, and I think she swallows more readily; the exudation was still quite profuse, but peeled off easier. I continued same remedies.

*November* 13*th,* 10 *o'clock A.M.*—Louisa about the same; think she paid more attention to her surroundings; there was not as much exudation; the throat looked pale and flabby; brushed it with the persulphate of iron, and continued same treatment.

*November* 14*th,* 10 *o'clock A.M.*—Louisa not so well; her nurse left yesterday, and things had returned to their old status. There was no increase of swelling nor exudation; she was sinking from exhaustion; there was a good deal of capillary congestion, with coldness of the extremities and surface; pulse barely perceptable at the wrist; put her into a warm bath and then wrapped her up in hot, dry flannels; substituted the whiskey for the wine. Continued the quinine, iron, and broths.

*November 15th.*—Louisa died last night at 10 o'clock.

CASE VII.—*November 17th*, 1864, was called to see David, a brother to Levi and Louisa, aged 11 years, also of good constitution and previous good health. He was taken with a chill in the night, and now had high fever, quick pulse, dry skin, with severe headache; he also complained of stiffness and soreness of the muscles of the neck and throat; considerable pain when swallowing; tonsils were swelled, and red with patches of exudation. Cauterized his throat freely with nitrate of silver, and left him quinæ sulph. grs. xx, pulvis Doveri, grs. vj, divided into six powders, one to be taken every four hours; also 15 drops of muriated tincture of iron, every four hours, in two tablespoonfuls of bourbon whiskey; steam the throat often and thoroughly with the fumes of strong vinegar and sage; rub the skin well with kerosene, and then apply a thin slice of salt pork about the neck.

*November 18th*, 10 *o'clock A.M.*—David decidedly worse; neck and face badly swollen; skin not so hot, but dry; pulse 110, quick and full; tonsils and fauces thoroughly covered with thick, bad looking and closely adhering exudation; he insisted that his throat had pained him more, and that he had had much more difficulty in swallowing ever since I cauterized it, yesterday; the kerosene and salt pork were bringing out a full eruption about the neck; pulse 120 per minute and very quick; respiration labored; deglutition quite difficult and painful; the exudation was very tough and closely adherent. Washed his throat with the kreosote mixture and continued the other treatment.

*November 19th*, 10 *o'clock A.M.*—David no better. Looked and felt bad that morning. Had that same sallow, sad, lifeless, tallowy look, skin was dusky and shining, similar to what we frequently see in low forms of pyemia, neck and face badly swollen, respiration quick and labored, deglutition difficult and painful, mouth and throat full of membrane, which could be peeled off quite readily in large flakes or pieces. The membrane had a peculiar dark and disorganized appearance, was very brittle. No fetor. Pulse 130, quick and feeble. The throat, where the membrane peeled off, was of a pale blue, and flabby appearance. The tonsils and fauces bled quite freely upon the slightest touch. After removing as much of the exudation as I could conveniently, I washed his throat with the dilute persulphate of iron, ordered two tablespoonfuls of bourbon whiskey every two hours, and continued the other remedies.

*November 20th, 10 o'clock A.M.*—David was covered all over with a peculiar erythematous redness, simulating at least scarlatina rash. He was as red as scarlet. There was no augmentation of heat. Pulse 126, slower and fuller. Swelling about neck and face subsiding—kerosene and pork had blistered him from ear to ear—respiration slower and easier, deglutition less painful, exudation not so profuse, throat and roof of mouth pale, and ragged looking. Washed them with the kreosote mixture, substituted warm poultices for the kerosene and pork, and continued the other treatment.

*November 21st, 10 o'clock A.M.*—David better. Redness disappearing. He looked and appeared decidedly better. Swelling subsiding rapidly, skin cool, soft, and moist. Pulse 100, and fuller. Exudation less, respiration and deglutition improved. He complained of being tired. Added 15 drops of the syrup of iodide of iron; continued treatment.

*November 22d.*—David improving finely. Redness all gone, skin moist and soft, swelling pretty much gone, no exudation, respiration and deglutition good. Reduced the quantity of whiskey one half, and continued other medicines.

*November 24th, 2 o'clock P.M.*—David doing finely. Bolstered up in bed, enjoying himself. Pulse 96, soft and regular. Appetite improving, bowels regular. I advised a continuance of the medicines in reduced quantities, and at longer intervals, for several days, and left him. I have seen his father frequently since, and learned from him that David finally got up and around, after a very slow and protracted convalescence.

Case VIII.—*November 12th*, 1864.—Was called to see Betsy H., a medium sized, well developed German girl, aged 16 years, a cousin to the above described patients, who attended the funeral of M——, on the 6th, a cold, rainy day. The services were held at the house, and she was in the house during the same, and then, in going from the house to the grave, and thence home, she became very cold and wet. She had been quite indisposed ever since. Yesterday she had a hard chill, followed with considerable fever. That morning she complained of headache, and sore throat, so I was sent for. I found her with high fever, flushed countenance, dry skin, stiffness and soreness in the muscles of the neck and throat, and some difficulty in swallowing. Upon inspecting the mouth and throat, I found the tonsils and fauces swollen, red, and covered with minute points of diphtheritic exudation. Pulse 100, quick and full, bowels regular. I washed her throat with the kreosote mixture, and left her quinæ sulph. grs. xxx, Doveri pulvis, grs. x, divided into five powders, and ordered that one be given every three hours; also two tablespoonfuls of bourbon whiskey and twenty drops of muriated tincture of iron every four hours; steam the throat with fumes of strong vinegar and sage, rub the skin about the neck thoroughly with kerosene, and then apply a thin slice of salt pork around the neck. Made her a weak solution of persulphate of iron to use as a gargle.

*November* 13*th*, 2 *o'clock P.M.*—Betsy is better. Her folks are very intelligent, and her mother an excellent nurse, who had followed all the directions to the letter, and, in consequence, her daughter was much better. Swelling about the neck and face less, fever abating, and she had less soreness and stiffness of the muscles of the neck, less difficulty in swallowing. There was an increased amount of exudation, but it was thin, and rubbed off quite readily. Pulse 96 per minute, full and soft. Washed her throat again with the kreosote mixture, and continued the other medicines.

*November* 14*th*, 2 *o'clock P.M.*—Betsy much better. Swelling about face and neck pretty nearly disappeared, swelling and redness of tonsils subsiding rapidly. Pulse 90, soft and full. Advised her to continue the medicines a day or two longer, and discharged her.

Although this patient recovered from the diphtheritic attack so readily, her health has been very poor ever since. She complains of extreme prostration, and great want of energy. Her catamenia have not made their appearance, although two periods have passed since. She looks quite pale and exsanguinated, and is still taking tonics and iron.

Case IX.—*November* 18*th*, 1864.—Was called to see Willie H——, a stout chunk of a German boy, aged 8 years, a brother to Betsy. He had not had any distinct chill, that they were aware of, but had complained a day or two of stiffness and soreness in the muscles of his neck. To-day, he seemed quite indisposed, and said that he could not swallow. Upon inspecting his mouth and throat, I found the tonsils and uvula swollen, and very red, and the left tonsil was well covered with diphtheritic exudation. Pulse 98, slow and full. Respiration normal, deglutition somewhat difficult and painful, bowels constipated. Washed the throat well with the kreosote mixture, and left him quinæ sulph. grs. xv, Doveri pulvis, grs. v, divided into four powders, one to be given every four hours; also a tablespoonful of bourbon whiskey, and ten drops of muriated tincture of iron, every four hours. Steam the throat well and often with the fumes of strong vinegar and sage, and apply the kerosene and salt pork externally to the neck, and left him a dilute solution of the persulphate of iron to use as a gargle.

*November* 19*th*, 3 *o'clock P.M.*—Willie better. There was but little external swelling. The tonsils were enlarged and red, and the left one covered to a considerable extent with exudation. Pulse 92 per minute, soft and full. Skin warm and moist, bowels moved twice since my last visit. He had but little thirst, no appetite, respiration easy, deglutition painful. He still complained of a good deal of soreness and stiffness of the neck. Notwithstanding the apparent mildness of the symptoms in this case, there was great muscular prostration, and much more fetor than in either of the above cases. Washed his throat with the kreosote mixture, and continued the other treatment.

*November* 20*th*, 3 *o'clock P.M.*—Willie was decidedly better. Found him bolstered up in a rocking-chair. Swelling and redness of tonsils subsiding, exudation mostly disappeared, no fever, appetite improving, bowels regular, skin soft, warm, and moist. Pulse 76, full and soft. Advised a continuance of the medicines in reduced quantities, and at longer intervals, for a few days more, and discharged him. This patient, notwithstanding the mildness of his attack, had a slow and protracted convalescence.

CASE X.—*November* 22*d*, 1864.—Was called to see Ellen, a stout, healthy looking German girl, aged 12 years, and a sister to Betsy and Willie, who had been complaining for several days of soreness and lameness all over her. Last night she had a slight chill, which was followed by fever, headache, and sore throat. I found her with fever, dry hot skin, and slight swelling about the angles of the jaw, face, and neck. Upon examing her throat, I found it inflamed, tonsils swelled, red, and studded with patches of diphtheritic exudation. Pulse 90 per minute, quick and full. Respiration normal, deglutition considerably painful, bowels have not moved in two days. Brushed her throat freely with the kreosote mixture, and left her quinæ sulph. grs. xx, Doveri pulvis, grs. x, divided into five powders, one every four hours; also two tablespoonfuls of bourbon whiskey and fifteen drops muriated tincture of iron every four hours. Steam her throat thoroughly with the fumes of strong vinegar and sage. Also left her a weak solution of persulphate of iron, to use as a gargle. Apply the kerosene and salt pork to the neck.

*November* 23*d*, 10 *o'clock A.M.*—Ellen decidedly better. Swelling of face and neck less, no fever, sweat freely last night. Pulse 86 per minute, slower and softer. Skin warm and moist, swelling of tonsils subsiding, exudation nearly disappeared, respiration normal, deglutition easy. Washed the throat with the kreosote mixture, and advised the continuance of the medicines for a day or two longer, and discharged her. This patient made a rapid and perfect recovery.

CASE XI.—*November* 10*th*, 1864.—Was called to see Mariah, another stout, healthy looking German girl, aged 8 years, of good constitution and previous good health. The mother informed me that this girl had been out several days in succession, husking corn, while the weather was very cold, and that she had become very cold and thoroughly chilled. At first she complained of a great deal of stiffness of her limbs, and soreness of the muscles all over her. For two days she had had headache, with stiff neck, and difficulty in swallowing. Her mother said that she had not noticed that she had had much fever. Bowels had been regular, and appetite pretty good. She said that she did not think her girl very sick, but thought, as I was passing by, she would call me in and have me see her. Upon inspecting her throat and mouth, I found the tonsils moderately swollen and inflamed, fauces red and dry. Upon the posterior surface of the right tonsil I found a sort of fissure, or ulcer, I should think half an inch long by several lines in breadth, which looked like a sort of indulent ulcer, and was covered and filled with diphtheritic exudation. There were also several other minute points of exudation surrounding this fissure upon the right tonsil. No fetor. Pulse quiet, and 82 to the minute. I cauterized the ulcer-like looking fissure with the solid nitrate of silver, and ordered that she steam her throat freely with the fumes of strong vinegar and sage, and that she gargle it thoroughly and often with a saturated solution of chlorate of potassa. Applied the kerosene and salt pork, and left her quinæ sulph. grs. xv, Doveri pulvis, grs. vj, divided into five powders, with orders that one be given every four hours.

*November* 11*th*, 11 *o'clock A.M.*—Found Mariah sitting up in the rocking-chair by the stove, feeling much better. Had slept well during the night, and sweat freely. Throat looked better, ulcered fissure looked cleaner and healthier; cauterized it again, and advised a continuance of the gargle, and occasional steaming. Repeated the quinine and Dover's powders, recommended that she be kept in the house and warm, and discharged her.

*November* 18*th*, 11 *o'clock A.M.*—Was called again to see Mariah. Found her in bed, face flushed, skin dry and hot, eyes suffused, and face and neck moderately swollen. Her mother informed me that after my last visit she improved, and they thought her entirely well until the day previous, when she began to complain of being chilly, and her head aching. She had been restless all night, and that morning complained of stiffness and soreness of her neck and throat. Upon inspecting her mouth and throat, I found the left tonsil and a portion of the left side of the roof of the mouth up to, and around, the uvula thickly covered with tough, nasty looking, closely adhering diphtheritic exudation. No fetor, respiration a little hurried, deglutition painful. Pulse 105 per minute, quick and jerking. The right tonsil was but little swelled, and the fissure entirely healed, no exudation upon it. Washed her throat with the kreosote mixture, ordered it steamed with the vinegar and sage, and gargled with a weak solution of the persulphate of iron. Applied the kerosene and salt pork, and left her quinæ sulph. grs. xv, Doveri pulvis, grs. vj, divided into five powders, with orders that she take one every four hours; also 15 drops of muriated tincture of iron in a tablespoonful of bourbon whiskey every four hours.

*November* 19*th*, 11 *o'clock A.M.*—Mariah no better. Looked and felt bad. Rested but little during the night. Considerable fever. Pulse 115 per minute, quick and irritable. Extensive swelling of the left side of the face and neck, skin dry, hot, and rough. Had considerable thirst, and a portion of the fluids attempted to be swallowed were rejected through the nose. Left side of throat, and posterior portion of left side of mouth were filled with bad looking exudation, which began to peel off a little. No fetor. She complained of feeling very tired and prostrate. No appetite. Removed as much of the exudation as I could, and washed the throat with the kreosote mixture, increased the whiskey to two tablespoonfuls every four hours, and continued the other treatment.

*November* 20*th*, 11 *o'clock A.M.*—Mariah no better. Swelling externally increasing. Kerosene and pork had caused a free pustulation. Respiration and deglutition difficult and painful in consequence of the extreme swelling. There was less heat of skin, but she began to show that same sallow, lifeless, tallowy appearance. Pulse 130 per minute, quick but feeble. Left tonsil and left side of mouth and throat still badly swollen, and thickly covered with dry, dark looking exudation. The exudation was dark colored and brittle, which peeled off in thick chunks. No fetor. Surface beneath the exudation pale blue, and bled quite freely. Removed as much of the membrane as I could conveniently, and then washed the throat with the persulphate of iron solution. Ordered the whiskey to be given every two hours, and added fifteen drops of the syrup of iodide of iron every four hours.

*November* 21*st*, 11 *o'clock A.M.*—Mariah about the same in all respects. Continued treatment.

*November* 22*d*, 11 *o'clock A.M.*—Mariah full as well. I think the swelling has subsided a little, and perhaps she swallowed a little easier. Pulse 132 and feeble; skin cool and blue; not much thirst. Bowels had moved twice since my last visit. Exudation a little diminished, but looked ragged and bad. No fetor. She had a peculiar abhorrence for chicken broth and beef tea, therefore, we had to feed her on milk porridge and gruel. Continued treatment.

*November* 23*d*, 11 *o'clock A.M.*—Mariah about the same; she seemed to be holding her own remarkably well. No increase of swelling; exudation less, and I think her throat looked better. The kerosene and pork had blistered her freely, therefore, we omitted them, and applied a warm poultice in their stead. Continued the other treatment.

*Nov.* 24*th*, 11 *o'clock A.M.*—Mariah looked and felt better; slept pretty well during the night; skin warmer and better color; no fever; pulse 128 per minute, and very feeble; respiration and deglutition improving; takes her food more readily; throat looked better; there was still considerable exudation, but it looked better. Continued all the treatment ordered at my last visit.

*November* 25*th*, 11 *o'clock A.M.*—Mariah improving; swelling was subsiding rapidly, and she was better in all respects. Continued treatment.

*November* 26*th*, 11 *o'clock A.M.*—Mariah still improving nicely. Continued treatment.

*November* 27*th*, 2 *o'clock P.M.*—Found Mariah quite comfortable; had rested well, and looked and appeared bright; no fever; skin soft and moist; pulse 98, slow and full; swelling about the face and neck nearly gone; exudation pretty much disappeared; throat pale, ragged, and flabby; appetite improving; washed the throat with the kreosote mixture; and continued the other treatment.

*November 28th*, 5 *o'clock A.M.*—Was summoned to visit Mariah, in haste, whom the messenger said was bleeding profusely from the mouth and nose. When I arrived at her house, I found things in a terrible plight: washdish, bedchamber, sheets, pillows, and towels all covered and saturated with blood. Her parents informed me that she first began to bleed about nine o'clock in the evening. She bled quite profusely from the nose at first, for a while, and then it stopped. Next she voided her urine, and it was mixed with blood; next she went to stool, and passed quite a quantity of blood from the bowels; soon thereafter she vomited, and also threw considerable blood from the stomach. She passed blood from the bladder and bowels several times during the night. The bleeding from the nose and mouth recommenced and continued without interruption until my arrival. The night had been an extremely dark and rainy one; the roads were in an almost impassable condition; and they residing eight miles from the city, did not send for me until daylight in the morning, when I found things as above described.

Mariah was lying among the bloody goods, pulseless at the wrist, very pale, bloodless, and faint. She could not articulate above a whisper, and complained of being very faint. She insisted that she must go to stool, but the least effort to move her, or place her upon the bedpan, would cause her to faint and convulse. I immediately brushed the throat and nostrils with the persulphate of iron, which arrested the bleeding immediately. I had her wrapped up in hot, dry flannels, and tried to crowd the stimulants, but all to no purpose. She grew extremely restless, and sank and died at 1 o'clock P.M.

The blood in the vessels, when I arrived, was in a fluid state. There was no effort at coagulation; it was very thin and dark colored, and remained so. There seemed to be an entire disorganization of the blood, and it oozed from all the mucous membranes; even the tears from the eyes were mixed with blood, and stained the towels and clothes. This is the only case that I have ever met of such extreme bleeding. I heard of one other, and that occurred in the Spring, in a boy five years old, belonging to the nearest neighbors of this same family. The father of the boy was there, and said that his boy had acted and died very much as this girl had.

Remarks.—It seems to me there can no longer be any question of its being a constitutional, or blood disease. I regard it as a zymotic disease of an adynamic or asthenic character, whose poison is most acutely debilitating, and seems to destroy or arrest that peculiar stimulus so necessary to excite and perpetuate the various vital phenomena, with a peculiar predisposition to local manifestations. This local manifestation, or exudation, I regard as an adventitious tissue pathognomonic of, and a consequence of the disease. It is most liable to occur in young persons—that period of life when the nutritive function gives a preponderance to the protien constituents of the blood, and inflammations which are so commonly attended with effusions of plastic or albuminous matter occur—hence its predisposition to this peculiar exudation. I say the natural tendency of the age is inclined to plastic effusions or exudations, but the disease in diphtheria has changed the blood so that its products have become aplastic, and, consequently, we have an alkaline and aplastic condition of the system. Is diphtheria a contagious or an infectious disease? This, I regret to say, is still an unsettled question. Leading minds in our profession differ widely upon this point. I have always regarded it as infectious. I am still of that opinion. There is no doubt to my mind but what the disease has been, and can be, communicated under certain circumstances. These circumstances are a continued or oft-repeated exposure in an illy-ventilated apartment where the air is humid, and it and the apartment thoroughly impregnated with the exhalations of the sick. Indeed, it is well known that no circumstance operates more extensively in favoring the speed, or in increasing the virulence and malignancy of all contagious or infectious diseases, than an impure and humid atmosphere. Its debilitating influences predispose to zymotic disease. It effects this in two ways. In the first place, it weakens the body, and thus acts as a true predisposing cause. And, in the second place, it leads to the concentration and increased virulence of the poison itself—not only by its general depressing agency, but also by impairing the condition of the blood itself, and by increasing in it the quantity of azotized matter in an incipient stage of corruption, which serves as a convenient nidus for the propagation of the diphtheritic poison. Again, upon the other hand, where the apartments are dry, well-warmed, ventilated, and kept rigidly clean, we have those poisonous emanations and exhalations so diluted and destroyed by the fresh and pure atmosphere that the danger of communicating or contracting the disease is materially lessened. This I deem an important consideration in the management of this disease, and one that well merits the early attention of the medical attendant. I cannot help but think that if this all important hygienic measure was rigidly enforced and thoroughly explained to patients, their friends, and the public, we should largely reduce the percentage of mortality in this disorder.

In all these cases, the predisposing causes must be referred to a morbific poison already present in the system—either accidental or congenital.

I think I have now fully stated my views of its predisposing causes. What the exciting causes are, I think is not so well understood nor clearly demonstrated. Experience has proved them liable to become operative at all times and under almost all circumstances. I have no doubt of their being endemic and extrinsic.

TREATMENT.—Few diseases, I apprehend, have been subjected to as many modes and varieties of treatment as diphtheria. Many prominent and worthy practitioners of medicine regard it as a local disease, requiring local treatment only. This class again divide, and differ widely in the selection of their remedies. Another class, equally prominent, intelligent, and worthy, regard it as a constitutional disease, requiring constitutional treatment. But they too differ equally as much in the selection of their remedies. This speculation and difference of opinion convinces me that the pathology of the disease is not fully understood, and that, as yet, no certain specifics have been found with which to arrest the disease. My notions are that the first class of the above-named physicians are greatly in error, and must of necessity be unsuccessful in their efforts and practice, so long as they regard and treat it as a local disorder. I fear, also, that many even of those who recognize the disease as a general or constitutional disease, still pay too much regard to the local complication, and treat the consequence more than the disease.

I am not satisfied that my patients have derived much benefit from local applications to the throat in diphtheria. When the local difficulty is excessive, and there is a tendency to, or danger of, sloughing and gangrene, then, of course, we should be on the lookout, and prevent such a result if possible. However, this condition of things I regard as quite unusual and accidental, and to be avoided by other and constitutional means. Of the various remedies recommended and made use of, I have been better satisfied with the effects of the alcoholic solution of kreosote than with any other article that I have used. I think its antiseptic properties recommend it peculiarly to correct the septic tendency of this condition, while its stimulating qualities give tone to the tissues, and prevent them from sloughing. Where the sloughing has already commenced, and the parts are pale, ragged, and flabby, I have used a solution of the persulphate of iron, and have been highly pleased with its effects. It is a powerfully tonic astringent, and as such constringes and hardens the tissues, reduces the swelling, lessens the effusion, and arrests the inflammatory tendency.

Nitrate of silver has not proved of service in my cases. *I am satisfied that severe cauterizations with nitrate of silver, or other caustics, in diphtheria, do more harm than good.* Such has been my experience.

Counter-Irritation.—Notwithstanding the great weight and majority of authorities are against the use of counter-irritants in diphtheria, I am in the habit of resorting to them early, and am convinced that my patients are benefited by mild and continued counter-irritation about the neck, and over the upper part of the chest. I think they act as revulsives, and detract or divert from the internal local tendency. I have never used cantharides, nor any of the other more powerful vesicating counter-irritants. I think that stimulating liniments and applications are preferable. I have generally made use of kerosene to rub the skin with, and applied a thin slice of bacon or salt pork. They are usually convenient, and generally ready at hand in every house—and I have been well pleased with their action and effects. They are always mild but certain, and I have never known them to prove painful, nor very annoying to the patient. They always produce a free pustular eruption, and I have never known any bad effects to follow their use in diphtheria.

General or Constitutional Treatment.—What should it consist of? That is the question! Let us examine for a moment what the indications are. What lesion have we to remedy? We have decided that the disease is a blood disease of an undoubted adynamic and asthenic character. Its first decided impression, in all severe and malignant cases, is upon the nervous centres, as is denoted by the general depression of all the vital phenomena of life. Next, we have a poisoned condition of the blood, which is increased as the disease advances, as is shown by the vascular prostration and excitement of the circulation. The rapid, quick pulse indicates that nature is putting forth an extra effort to rid the economy of the poison. In many of the milder cases she succeeds unaided, but in many of the severer and more malignant cases she fails for the want of power. Nature is exhausted. The depressed condition of the nervous system, and the poisoned condition of the blood have absorbed and used up the vital stimuli necessary to excite and perpetuate the animal functions, and the patient sinks and dies from exhaustion—dies for the want of steam and power to propel the machinery of life. Now, what are the indications? What is to be done? How is the catastrophe to be avoided? Most obviously by eliminating the poison and supplying the material for steam and power. What does that material consist of, and where is it to be sought? I answer among the tonics, stimulants, and nutriments. The next question, then, is what articles of the materia medica offer the greatest inducements? Which contain the greatest number of the above requirements? I cannot think of any one article that offers so great inducements as alcohol and its preparations. It ranks as chief among the general stimulants; under its influence the heart and arteries pulsate more strongly, the capillaries become distended with red blood, the animal temperature is raised, and all the functions, but those of the nervous system in particular, are rendered more active. But the benefits of alcoholic medicines are not limited to their stimulant influence. They are also food, according to the present chemical doctrines, in the sense that they restrict the waste of the body, and therefore indirectly sustain the strength, that is to say, they contain and afford a large quantity of hydrogen, with which the inspired oxygen combines instead of with the tissues themselves. They are also absorbed and enter the blood itself, and are thus carried everywhere through the system to arouse the dormant powers, excite a craving for food, quicken its digestion, promote its assimilation, and thus become, as it were, purveyors to the nutrition of all the organs. Therefore I claim it to be peculiarly adapted to the treatment of these low and malignant cases of diphtheria. Of the many alcoholic preparations, I have preferred and used the bourbon whiskey. I have commenced its use early in the disease, and continued it in large doses for days in succession, as I think, with the happiest results. I think it should be given in quantities sufficient to make and maintain a decided impression on the system. Not sufficient to produce a sedative effect, but enough to stimulate and keep up the functions and hold them firm until the poison can be eliminated and thrown off. It will be noticed that of the eleven cases above reported, nine took whiskey largely through their entire sickness. Eight of the nine recovered. The ninth was improving decidedly and rapidly, when the hemorrhage of which she died occurred. I have every reason to believe that she too would have recovered, had not the hemorrhage occurred.

*Two of the eleven took no whiskey, and both died.* I do not think that the disease in their cases was any more severe or malignant in its commencement than in the others. Their surroundings also were the same. They all occurred in one neighborhood, covering a space of country two miles in width by six miles in length. Their parents were all prairie farmers. The land is low, and has a clay and loam subsoil. In ordinary, and in wet seasons a considerable portion of the prairie is covered with water; but the past season having been a very dry one in this section, their farms were dry, and they had an unusual growth of vegetation.

Mr. President and Gentlemen, although one of the leading ideas of this article is to suggest the adaptation, and urge the utility of whiskey in diphtheria, I must not be misunderstood as relying upon it alone. I understand too well the qualities and benefits of sulphate of quiniæ, muriated tincture of iron, hydrochlroic acid, and the iodide of iron, in all this class of diseases, to reject them in diphtheria. They are all entitled to our utmost confidence, and should be early and energetically administered in this disease—both as prophylactics and curatives. I say, give plenty of fresh, dry air; bourbon whiskey, largely; quiniæ; muriated tincture of iron; hydrochloric acid; iodide of iron, with the best of nourishment internally; with mild but continued counter-irritation externally; *cleanliness imperative.*

